# Association of Triglyceride–Glucose Index With Different Cardiovascular Diseases in Non‐Diabetic Hypertension

**DOI:** 10.1111/jcmm.70925

**Published:** 2025-10-30

**Authors:** Qiyu Xiao, Xiaoying Li, Xi Zhou, Zhengbin Yao, Rui He, Yong Long, Yingjie Su

**Affiliations:** ^1^ Department of Nuclear Medicine, The Affiliated Cancer Hospital of Xiangya School of Medicine Central South University/Hunan Cancer Hospital Changsha Hunan China; ^2^ Department of Emergency Medicine, The Affiliated Changsha Central Hospital, Hengyang Medical School University of South China Changsha Hunan China

**Keywords:** cardiovascular disease, hypertension, peripheral arterial disease, stroke, triglyceride–glucose index

## Abstract

This research intends to explore the association of the triglyceride‐glucose (TyG) index with cardiovascular disease (CVD) death, stroke, myocardial infarction (MI), heart failure (HF) and peripheral arterial disease (PAD) in non‐diabetic hypertension. This post hoc analysis uses data from a large‐scale randomised controlled trial (RCT) study—Systolic Blood Pressure Intervention Trial (SPRINT), and we used Cox proportional hazards regression to explore the relationship between TyG and different CVDs. This longitudinal analysis of 9323 participants revealed a significant positive association between the TyG index and CVD death (HR 1.91, 95% CI 1.34, 2.73), HF (HR 1.43, 95% CI 1.05, 1.94), MI (HR 1.30, 95% CI 1.00, 1.69), stroke (HR 1.60, 95% CI 1.16, 2.21) and PAD (HR 1.78, 95% CI 1.30, 2.44). This positive correlation was consistently observed across different subgroups. Trend tests were significant for CVD death, heart failure, MI, stroke and PAD (*p* < 0.05). In non‐diabetic hypertensive patients, a higher TyG index was associated with a higher risk of CVD (CVD death, stroke, HF, MI, PAD).

## 1 Introduction

Globally, cardiovascular disorders (CVD) represent the primary aetiology of both incidence and mortality rates across populations, presenting a significant population wellness concern and placing an economic strain on affected individuals [1, 2, 3, 4]. The contributing factors to CVD, including dyslipidaemia, smoking, hypertension, obesity and type 2 diabetes mellitus (T2DM), are well‐established and clinically managed [5, 6]. Notably, T2DM and prediabetes are prevalent in patients diagnosed with CVD and are associated with poor prognosis [7, 8, 9]. However, research has found that some individuals still develop CVD, even when these risk factors are well managed or in the absence of these common risk factors [10, 11]. Therefore, identifying residual cardiovascular risk factors is crucial for controlling the occurrence and progression of cardiovascular diseases.

Insulin resistance (IR), which refers to an impaired ability of cells to respond normally to insulin and utilise it for metabolic processes, is considered a key feature of T2DM, often present even before diabetes is clinically diagnosed [12, 13, 14]. Accumulating evidence suggests that IR and related conditions are crucial for the onset and progression of CVD in both individuals with diabetes and those without diabetes. It is widely recognised that IR predisposes individuals to various metabolic disturbances, ranging from hyperglycaemia to dyslipidaemia and hypertension, all of which are closely linked to adverse cardiovascular outcomes [14, 15]. The TyG index—a mathematical function based on fasting triglyceride and glucose levels (Ln[TG × Glu/2] in mg/dL)—serves as a composite biomarker for assessing IR [16]. Although previous studies have linked the TyG index to CVD, evidence in non‐diabetic hypertensive adults remains scarce. Therefore, this study will use baseline and follow‐up data from the Systolic Blood Pressure Intervention Trial (SPRINT) to comprehensively examine the independent effect of the TyG index on CVD death, heart failure (HF), stroke, peripheral arterial disease (PAD) and myocardial infarction (MI) in this population.

## 2 Materials and Methods

### 2.1 Data Source and Study Participants

The SPRINT study is a large‐scale randomised controlled trial (RCT) that evaluated the comparative clinical benefits of a stringent (< 120 mmHg) versus a standard (< 140 mmHg) systolic blood pressure target in a large cohort of hypertensive patients without diabetes. Data from this study are available through the Biologic Specimen and Data Repository Information Coordinating Center (BioLINCC) of the National Heart, Lung and Blood Institute (NHLBI) at https://biolincc.nhlbi.nih.gov/home. A comprehensive account of the study's execution, prespecified clinical objectives and pertinent results has been provided in prior publications [17, 18]. The current analysis was conducted on data obtained from the aforementioned large‐scale RCT, following a post hoc analytical framework. The study initially enrolled 9361 participants, with 38 excluded due to missing baseline TyG index data, resulting in a final cohort of 9323 individuals.

### 2.2 Definitions of Triglyceride–Glucose Index and Clinical Outcomes

We chose the TyG index as the exposure variable and computed it using the formula: Ln [fasting triglycerides (mg/dL) × fasting glucose (mg/dL)]/2 [19, 20]. Quantification of serum triglyceride and glucose concentrations was performed via enzymatic assays utilising a fully automated biochemistry platform. Clinical events during follow‐up will be ascertained by collecting participant‐reported episodes, protocol‐mandated laboratory and ECG data, and classifying them blind to treatment assignment by the Morbidity and Mortality subcommittee; supplemental linkage to the National Death Index (NDI) will complete the data set. This study collected a total of five clinical outcome measures, which are CVD death, HF, stroke, PAD and MI. CVD death was defined as death due to fatal coronary heart disease, HF, stroke (ischemic or haemorrhagic) or other non‐coronary cardiovascular events such as aortic aneurysm rupture and arrhythmia. HF is defined as definite or suspected acute decompensated HF presenting with multiple signs/symptoms of cardiac decompensation/inadequate pump function, requiring hospitalisation or an emergency department visit with intravenous therapy. Stroke encompasses cerebral infarction, subarachnoid haemorrhage, intraparenchymal haemorrhage, other haemorrhages and unknown types. PAD events are defined as carotid or peripheral revascularisation, abdominal aortic aneurysm repair and other objectively documented PAD. MI is defined as myocardial necrosis resulting from coronary occlusion due to spasm, embolism, thrombus, plaque rupture. A detailed description of the definitions can be found in Table S1.

### 2.3 Covariates

The covariates included in this research were gender, age, race, smoking status, alcohol consumption status, vigorous physical activity, Framingham risk score, body mass index (BMI), medication use (aspirin, statins), comorbidities (CVD, chronic kidney disease, hyperlipidaemia) and anti‐hypertensive strategies. The specific definitions and criteria are detailed in Table S1.

### 2.4 Statistical Analysis

Individuals were segmented into four subsets corresponding to the quartiles (Q1–Q4) of the TyG index. We employed frequency counts (percentages) for categorical variables and applied the mean ± standard deviation for continuous variables. Differences in categorical data were assessed using *χ* ^2^ or Fisher's exact tests. *T*‐tests were applied to compare continuous variables across groups. Missing continuous variables (BMI and Framingham Risk Score) were imputed using median values, with missing counts of 62 (0.67%) and 5 (0.05%), respectively. Categorical variables with missing values (smoking status [*n* = 11, 0.12%], alcohol consumption [*n* = 72, 0.77%], vigorous physical activity [*n* = 32, 0.34%], aspirin use [*n* = 19, 0.20%], statin use [*n* = 61, 0.65%]) were assigned to a distinct category within the model. We established a multivariable Cox proportional hazards regression model, and by constructing two models with stepwise adjustment for confounding factors, the proportional hazards assumption is evaluated using the Schoenfeld residuals test. We examined the relationship between the TyG index and a spectrum of cardiovascular disorders. Model I adjusted for none. Model II adjusted for gender, age (continuous variables), race, smoking status, alcohol consumption, vigorous physical activity, BMI (continuous variables), CKD, aspirin use, statin use, hyperlipidaemia, CVD, Framingham risk score (continuous variables). TyG index quartiles were grouped to perform trend tests to explore the non‐linear relationship between the independent and dependent variables. Subgroup analyses were stratified by age (< 75 and ≥ 75), gender (male and female), race (white and non‐white), vigorous physical activity (< 1 time/week and ≥ 1 time/week), alcohol consumption (yes and no), smoking status (yes and no), CVD (yes and no), CKD (yes and no), hyperlipidaemia (yes and no). The R project (version 4.5.1) and EmpowerStats (www.empowerstats.com) were utilised for all statistical computations. Statistical significance was set at a *p*‐value < 0.05.

## 3 Results

Stratified by TyG index quartiles, the baseline demographic and clinical profiles of the study cohort were evaluated and reported (Table 1). A total of 9323 participants were included in our study. Among the participants, the mean age, BMI and Framingham risk score were 67.87 years, 29.85 kg/m^2^ and 17.38%, respectively. 57.77% were white, 13.28% were current smokers, 51.69% were alcohol consumers, 56.48% engaged in vigorous physical activity ≥ 1 time/week. The proportions of individuals using aspirin, statins, having CVD, hyperlipidaemia, CKD and assignment to intensive anti‐hypertensive therapy stood at 50.92%, 43.40%, 20.05%, 57.77%, 28.36%, 50.01%, respectively.

**TABLE 1:** Baseline characteristics of participants in the study by quartiles of the triglyceride–glucose index.

	Total	Q1 (*n* = 2330)	Q2 (*n* = 2327)	Q3 (*n* = 2335)	Q4 (*n* = 2331)	*p*
Gender						
Male	6016 (64.53%)	1443 (61.93%)	1454 (62.48%)	1525 (65.31%)	1594 (68.38%)	< 0.001
Female	3307 (35.47%)	887 (38.07%)	873 (37.52%)	810 (34.69%)	737 (31.62%)	
Age (years)	67.87 ± 9.41	69.31 ± 9.61	68.43 ± 9.57	67.94 ± 9.15	65.81 ± 8.92	< 0.001
Race						
White	5386 (57.77%)	1174 (50.39%)	1313 (56.42%)	1433 (61.37%)	1466 (62.89%)	< 0.001
Hispanic	978 (10.49%)	153 (6.57%)	212 (9.11%)	274 (11.73%)	339 (14.54%)	
Black	2785 (29.87%)	960 (41.20%)	769 (33.05%)	582 (24.93%)	474 (20.33%)	
Other	174 (1.87%)	43 (1.85%)	33 (1.42%)	46 (1.97%)	52 (2.23%)	
Smoking status						
Never smoked	4111 (44.10%)	1054 (45.24%)	1042 (44.78%)	1037 (44.41%)	978 (41.96%)	0.358
Former smoker	3963 (42.51%)	965 (41.42%)	988 (42.46%)	999 (42.78%)	1011 (43.37%)	
Current smoker	1238 (13.28%)	308 (13.22%)	293 (12.59%)	297 (12.72%)	340 (14.59%)	
Not recorded	11 (0.12%)	3 (0.13%)	4 (0.17%)	2 (0.09%)	2 (0.09%)	
Alcohol consumption						
No drinking	4432 (47.54%)	1113 (47.77%)	1134 (48.73%)	1129 (48.35%)	1056 (45.30%)	0.023
Drinking	4819 (51.69%)	1197 (51.37%)	1169 (50.24%)	1187 (50.84%)	1266 (54.31%)	
Not recorded	72 (0.77%)	20 (0.86%)	24 (1.03%)	19 (0.81%)	9 (0.39%)	
Vigorous physical activity						
< 1 time/week	4025 (43.17%)	975 (41.85%)	997 (42.84%)	1011 (43.30%)	1042 (44.70%)	0.177
≥ 1 time/week	5266 (56.48%)	1347 (57.81%)	1320 (56.73%)	1321 (56.57%)	1278 (54.83%)	
Not recorded	32 (0.34%)	8 (0.34%)	10 (0.43%)	3 (0.13%)	11 (0.47%)	
Body mass index (kg/m^2^)	29.85 ± 5.76	28.43 ± 5.91	29.47 ± 5.80	30.38 ± 5.72	31.13 ± 5.24	< 0.001
Aspirin use						
No	4557 (48.88%)	1148 (49.27%)	1121 (48.17%)	1099 (47.07%)	1189 (51.01%)	0.166
Yes	4747 (50.92%)	1176 (50.47%)	1202 (51.65%)	1230 (52.68%)	1139 (48.86%)	
Not recorded	19 (0.20%)	6 (0.26%)	4 (0.17%)	6 (0.26%)	3 (0.13%)	
Statin use						
No	5216 (55.95%)	1369 (58.76%)	1301 (55.91%)	1250 (53.53%)	1296 (55.60%)	0.007
Yes	4046 (43.40%)	942 (40.43%)	1008 (43.32%)	1070 (45.82%)	1026 (44.02%)	
Not recorded	61 (0.65%)	19 (0.82%)	18 (0.77%)	15 (0.64%)	9 (0.39%)	
Cardiovascular disease						
No	7454 (79.95%)	1867 (80.13%)	1862 (80.02%)	1857 (79.53%)	1868 (80.14%)	0.948
Yes	1869 (20.05%)	463 (19.87%)	465 (19.98%)	478 (20.47%)	463 (19.86%)	
Hyperlipidaemia						
No	3937 (42.23%)	1539 (66.05%)	1222 (52.51%)	941 (40.30%)	235 (10.08%)	< 0.001
Yes	5386 (57.77%)	791 (33.95%)	1105 (47.49%)	1394 (59.70%)	2096 (89.92%)	
Chronic kidney disease						
No	6679 (71.64%)	1738 (74.59%)	1662 (71.42%)	1646 (70.49%)	1633 (70.06%)	0.002
Yes	2644 (28.36%)	592 (25.41%)	665 (28.58%)	689 (29.51%)	698 (29.94%)	
Framingham risk score	17.38 ± 2.49	16.94 ± 2.38	17.18 ± 2.43	17.54 ± 2.53	17.87 ± 2.54	< 0.001
Antihypertensive strategies						
Standard treatment	4661 (49.99%)	1147 (49.23%)	1179 (50.67%)	1141 (48.87%)	1194 (51.22%)	0.312
Intensive treatment	4662 (50.01%)	1183 (50.77%)	1148 (49.33%)	1194 (51.13%)	1137 (48.78%)	

*Note:* Continuous variables are expressed as mean ± standard deviation and *p* values were calculated using *t*‐test. Categorical variables are expressed as frequencies (percentages) and *p* values were calculated using *χ* ^2^ or Fisher's exact tests.

**FIGURE 1:**
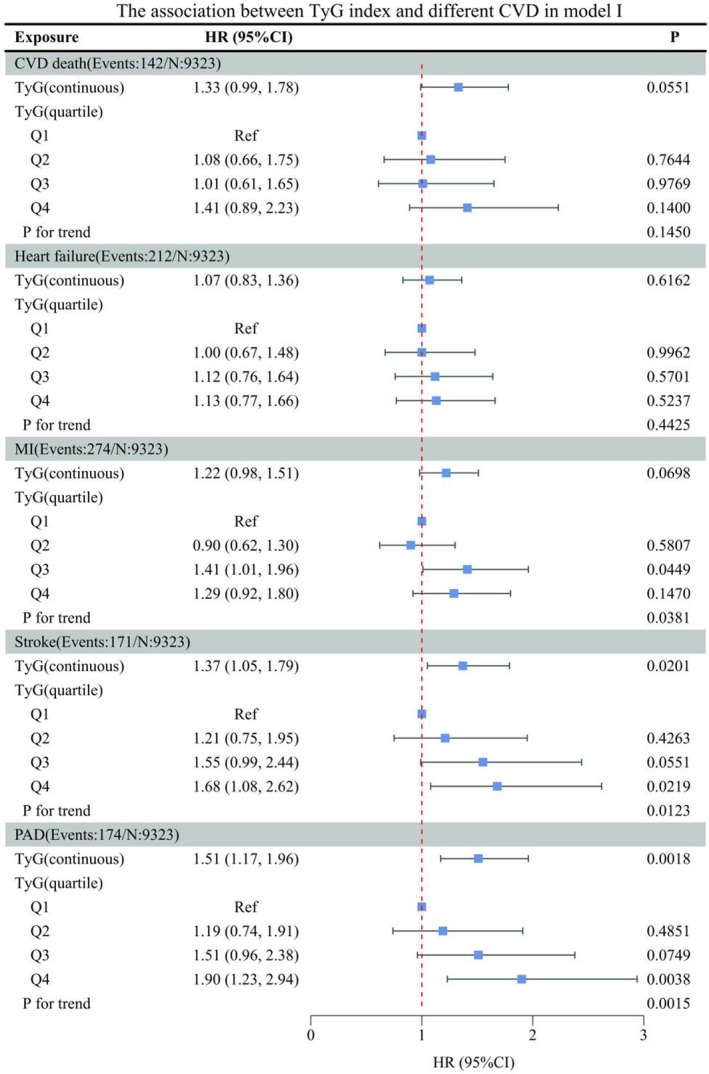
The association between TyG index and different CVD in non‐diabetic hypertension in model I. CI, confidence interval; CKD, chronic kidney disease; CVD, cardiovascular disease; HR, hazard ratio; MI, myocardial infarction; PAD, peripheral arterial disease; Ref, reference; TYG, triglyceride glucose. Adjusted for: None.

**FIGURE 2:**
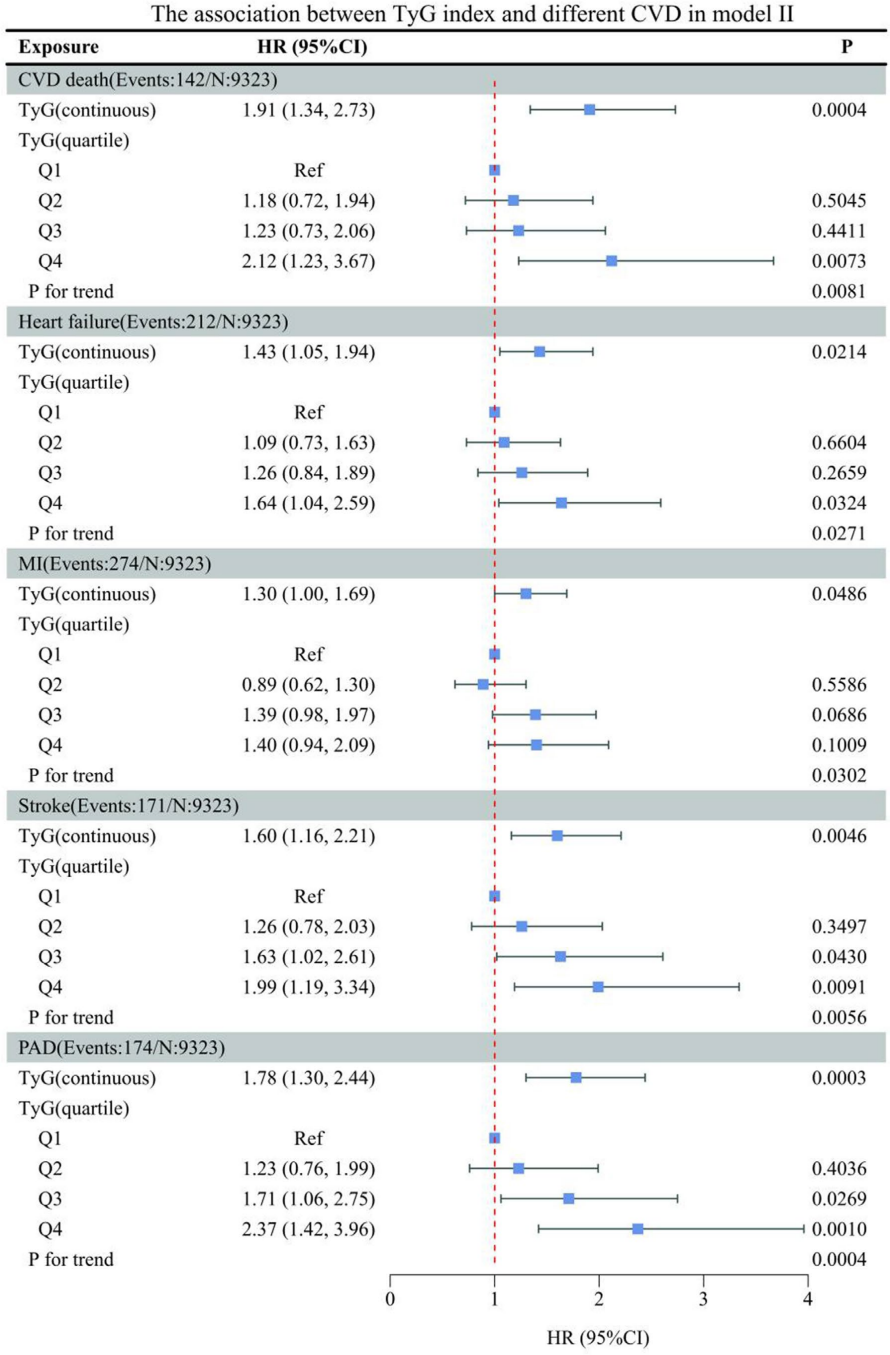
The association between TyG index and different CVD in non‐diabetic hypertension in model II. BMI, body mass index; CI, confidence interval; CKD, chronic kidney disease; CVD, cardiovascular disease; HR, hazard ratio; MI, myocardial infarction; PAD, peripheral arterial disease; Ref, reference; TYG, triglyceride glucose. Adjusted for: Gender, age, race, smoking status, alcohol consumption, vigorous physical activity, BMI, CKD, aspirin use, statin use, hyperlipidaemia, CVD, Framingham risk score.

**FIGURE 3:**
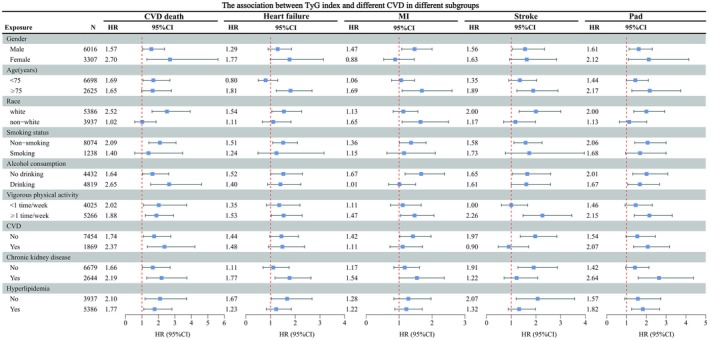
The association between TyG index and different CVD deaths in different subgroups. BMI, body mass index; CI, confidence interval; CKD, chronic kidney disease; CVD, cardiovascular disease; HR, hazard ratio; MI, myocardial infarction; PAD, peripheral arterial disease; TYG, triglyceride‐glucose. Adjusted for: Gender, age, race, smoking status, alcohol consumption, vigorous physical activity, BMI, CKD, aspirin use, statin use, hyperlipidaemia, CVD, Framingham risk score except the subgroup's variable.

In this research, to determine whether the TyG index is correlated with diverse forms of CVD, two models were developed by us, with Model I being unadjusted (Figure 1). A significant positive correlation was observed for both stroke (HR 1.37, 95% CI 1.05, 1.79) and PAD (HR 1.51, 95% CI 1.17, 1.96) when the TyG index was treated as a continuous variable. Subsequently, we performed trend tests after dividing the subjects into quartiles based on the TyG index, and significant trends were observed in MI, stroke and PAD (all *p* < 0.05). In Model II (Figure 2), we adjusted for the following variables related to socio‐demographics, lifestyle, medication use, comorbidities: gender, age, race, smoking status, alcohol consumption, vigorous physical activity, BMI, CKD, aspirin use, statin use, hyperlipidaemia, CVD, Framingham risk score. When using the TyG index in continuous form for regression analysis, it exhibited a significant and positive correlation with several cardiovascular outcomes, including CVD death (HR 1.91, 95% CI 1.34–2.73), HF (HR 1.43, 95% CI 1.05–1.94), MI (HR 1.30, 95% CI 1.00–1.69), stroke (HR 1.60, 95% CI 1.16–2.21) and PAD (HR 1.78, 95% CI 1.30–2.44). Additionally, trend tests revealed significant linear associations for CVD mortality, heart failure, MI, stroke, PAD (all *p* < 0.05). We next turned to subgroup analyses to investigate heterogeneity in the TyG–CVD associations (Figure 3). In different subgroups, TyG was positively correlated with the outcome indicators, except in the subgroup of HF aged < 75 years, the female subgroup of MI, the CVD (yes) subgroup of stroke.

**FIGURE 4:**
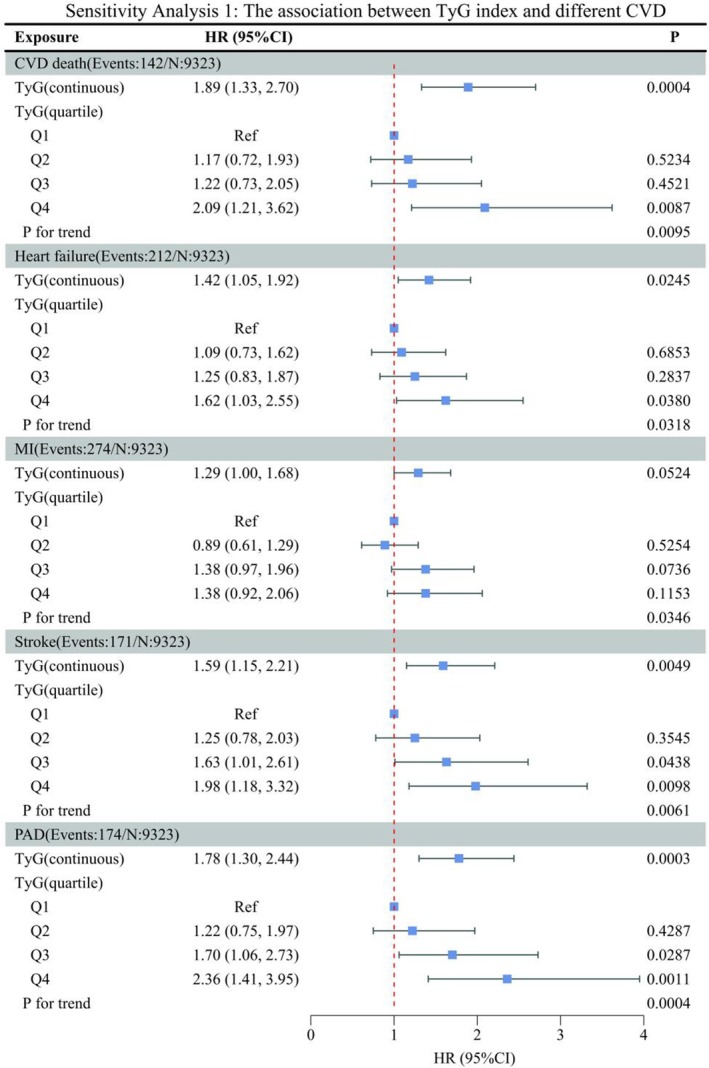
The association between TyG index and different CVD in non‐diabetic hypertension (*n* = 9323). BMI, body mass index; CI, confidence interval; CKD, chronic kidney disease; CVD, cardiovascular disease; HR, hazard ratio; MI, myocardial infarction; PAD, peripheral arterial disease; Ref, reference; TYG, triglyceride‐glucose. Adjusted for: Gender, age, race, smoking status, alcohol consumption, vigorous physical activity, BMI, CKD, aspirin use, statin use, hyperlipidaemia, CVD, Framingham risk score, antihypertensive strategies.

To assess the robustness of our findings, we performed comprehensive sensitivity testing: (1) Since the SPRINT study confirmed that intensive blood pressure‐lowering therapy reduces CVD risk, we further adjusted for the anti‐hypertensive strategies variable in the model (Figure 4). (2) To more accurately reflect the true level of TyG, TyG uses an average based on baseline values and re‐measurement over 2 years (Table S2). (3) To avoid reverse causality, we excluded individuals who experienced the outcome indicators within the first year (Table S3). We found that TyG was positively correlated with various CVD outcomes under different sensitivity analysis strategies.

## 4 Discussion

By conducting a secondary analysis of a major RCT's data set, we found that TyG is positively correlated with various cardiovascular outcome indicators (CVD death, stroke, HF, MI and PAD) in non‐diabetic hypertension. Moreover, as the level of TyG increases, the hazard of developing relevant CVD in the future is higher.

Currently, the gold standard for evaluating insulin resistance is the HOMA‐IR, derived from the homeostatic model assessment. However, its application value is limited in patients receiving insulin therapy or with pancreatic beta‐cell dysfunction [21]. In contrast, evidence suggests that the TyG index outperforms HOMA‐IR in detecting IR across individuals with or without diabetes [22]. Mechanistic and observational studies consistently suggest that the TyG index elevation may serve as a predictor for adverse cardiovascular events. Wang et al. revealed the capacity of TyG to serve as a significant indicator of CVD severity, especially in people with prediabetes [23]. Moreover, an analysis of 35,455 high‐risk CVD patients revealed an association between elevated TyG index levels and increased risk of all‐cause as well as cardiovascular mortality [24]. Notably, for future CVD risk, the TyG index has predictive value in patients with diabetes and those without diabetes [25, 26, 27]. A large‐scale cohort study enrolling approximately 100,000 Chinese participants (median follow‐up: 11.03 years) demonstrated that elevated baseline TyG levels and their long‐term persistence were associated with heightened MI risk [28]. In a prospective cohort of 12,374 individuals free of baseline HF or coronary heart disease (CHD), elevated TyG index levels were prospectively linked to incident heart failure and adverse cardiac remodelling [29].

Currently unclear is the precise mechanism by which TyG is linked to CVD. However, as an index composed of two cardiovascular risk factors (triglycerides and glucose), it reflects the level of an individual's IR. First, IR induces dyslipidaemia, marked by elevated low‐density lipoprotein cholesterol (LDL‐C) and reduced high‐density lipoprotein cholesterol (HDL‐C), along with impaired glucose metabolism (hyperglycaemia). These alterations, in turn, trigger inflammatory and oxidative stress responses, ultimately resulting in atherosclerosis [30]. Second, IR promotes aberrant nitric oxide (NO) signalling and triggers mitochondrial dysfunction via electron transport chain overstimulation, a key source of reactive oxygen species (ROS). The excessive levels of NO and ROS collectively drive endothelial dysfunction [31, 32]. Third, IR can lead to excessive platelet activation and simultaneously trigger the occurrence of thrombotic and inflammatory events by induction of platelet‐derived tissue factor via adhesion and thromboxane A2 (T × A2) signalling pathways [33]. Finally, IR promotes the proliferation of smooth muscle cells, collagen cross‐linking and collagen accumulation. These pathological changes can lead to cardiac diastolic dysfunction, myocardial fibrosis and ultimately result in HF [26]. Therefore, the TyG index can function as an alternative indicator for identifying IR or assessing high‐risk populations for diabetes in large‐scale studies. Moreover, it has good predictive power for CVD. Further research into its underlying mechanisms is warranted in the future. The current research on TyG also has certain limitations. First, most research indicators are based on TyG measured in the fasting state, while postprandial measurements of triglycerides and glucose levels are abnormal responses to IR. The potential clinical value of postprandial TyG remains unclear. Second, patients with CVD often present with elevated triglyceride levels and hyperglycaemia, and the causal relationship between TyG and CVD remains unclear. Third, the current study populations are mainly focused on middle‐aged and elderly patients, and current research exhibits a paucity of evidence regarding CVD risk prediction in younger populations.

The focus of our study is that this is a large, well‐conducted randomised controlled trial. This study included a cohort of 9323 participants, providing robust statistical power to advance understanding in this research domain. Furthermore, we mitigated confounding through comprehensive adjustment for covariates, enhancing result validity and generalisability across heterogeneous populations. Finally, to enhance the robustness of the TyG index–CVD association assessment, sensitivity analyses and subgroup stratification were systematically implemented. Although statistical significance varied for some endpoints (HF, MI, stroke) across sensitivity analyses, the direction of effect remained consistently positive (HR > 1) or trend tests were significant. So it still partially reflects the association between them. Despite the advantages of our study, there are some potential limitations that cannot be ignored. First of all, the TyG index is influenced by multiple factors such as lifestyle, diet and medications, leading to intra‐individual variability. Although a single baseline measurement has demonstrated predictive value in cohort studies, it may still misclassify an individual's long‐term metabolic status and potentially attenuate the observed associations. Second, because the study population comprised hypertensive patients without diabetes, our findings may not be generalisable to other groups. Third, the absence of data on fasting insulin levels precludes the current comparison of the TyG index with other gold‐standard measures of insulin resistance. Fourth, the Cox proportional hazards model used in this study does not account for competing risks from non‐CVD deaths. Therefore, our conclusions may have overestimated the true cause‐specific risk.

## 5 Conclusion

In conclusion, our study revealed that the risk of different cardiovascular diseases (CVD death, stroke, HF, MI, PAD) is higher in non‐diabetic hypertension with a higher TyG index.

## Author Contributions

**Qiyu Xiao:** conceptualization (equal), data curation (equal), writing – original draft (equal). **Xiaoying Li:** conceptualization (equal), formal analysis (equal), writing – original draft (equal). **Xi Zhou:** conceptualization (equal), investigation (equal), project administration (equal). **Zhengbin Yao:** formal analysis (equal), resources (equal), software (equal). **Rui He:** investigation (equal), methodology (equal), visualization (equal), writing – review and editing (equal). **Yong Long:** project administration (equal), validation (equal), visualization (equal). **Yingjie Su:** conceptualization (equal), writing – review and editing (equal).

## Ethics Statement

The SPRINT study was approved by the institutional review boards of the participating study sites, and all participants signed an informed consent form.

## Consent

The authors have nothing to report.

## Conflicts of Interest

The authors declare no conflicts of interest.

## Supporting information


**Table S1:** The definition of outcomes and covariates.

**Table S2:** The association between TYG and different CVD in non‐diabetic hypertension (*n* = 8109).

**Table S3:** The association between TYG and different CVD in non‐diabetic hypertension.

## Data Availability

The data sets used and/or analysed during the present study were availed by the corresponding author (Yingjie su) on reasonable request. The data can be downloaded from “https://biolincc.nhlbi.nih.gov/home”.
