# Supplementary material for: Association of Triglyceride–Glucose Index With Different Cardiovascular Diseases in Non‐Diabetic Hypertension
Source: J Cell Mol Med. 2025 Oct 30;29(21):e70925. doi: 10.1111/jcmm.70925 (PMC12573475; doi:10.1111/jcmm.70925)
Supplement: Supplementary file 1 — Table S1: The definition of outcomes and covariates. [file JCMM-29-e70925-s003.docx]

**Supplementary table 1**. The definition of outcomes and covariates.

| **Outcomes** | Definition |
| --- | --- |
| CVD | 1.Previous myocardial infarction, percutaneous coronary intervention, coronary artery bypass grafting, carotid endarterectomy, carotid stenting.  2.Peripheral artery disease with revascularization.  3. Acute coronary syndrome with or without resting ECG change, ECG changes on a graded exercise test, or positive cardiac imaging study.  4.At least a 50% diameter stenosis of a coronary, carotid, or lower extremity artery.  5.Abdominal aortic aneurysm ≥5 cm with or without repair.  6.Coronary artery calcium score ≥ 400 Agatston units within the past 2  years.  7. Ankle brachial index (ABI) ≤0.90 within the past 2 years.  8. Left ventricular hypertrophy (LVH) by ECG (based on computer reading),  echocardiogram report, or other cardiac imaging procedure report within  the past 2 years. |
| Peripheral arterial disease | PAD event defined as any of the following:  1. carotid angioplasty or endarterectomy.  2. peripheral angioplasty or thrombolysis (with or without stenting, including renal artery stenosis, subclavian artery stenosis, and femoral artery stenosis).  3. peripheral vascular surgery.  4. lower-extremity amputation (due to ischemia or gangrene).  5. Thoracoabdominal aortic aneurysm surgery or vascular surgery.  6. Other objectively defined PAD events. |
| **Covariates** | Definition |
| Alcohol consumption | No drinking is defined as less than one drink per month. |
| Vigorous physical activity | Vigorous physical activities are those that make you sweat, have a fast heartbeat, or breathe faster. |
| Hyperlipidemia | Participants with fasting Lipids that meet one of the following criteria will be defined as hyperlipidemic: 1. triglycerides ≥ 150 mg/dL, 2. total cholesterol ≥ 200 mg/dL, 3. low-density lipoprotein(LDL) ≥ 130 mg/dL, 4. high-density lipoprotein(HDL) ≤ 40 mg/dL for men and ≤ 50 mg/dL for women |
| Chronic kidney disease | Chronic kidney disease defined as GFR less than 60 mL/min/1.73m^2^. GFR was calculated by the Modification of Diet in Renal Disease equation. |
| BMI | BMI is calculated by dividing weight by the square of height. |

Abbreviations: GFR, glomerular filtration rate; CVD, cardiovascular disease; BMI, Body mass index.
