# Supplementary material for: Association of Triglyceride–Glucose Index With Different Cardiovascular Diseases in Non‐Diabetic Hypertension
Source: J Cell Mol Med. 2025 Oct 30;29(21):e70925. doi: 10.1111/jcmm.70925 (PMC12573475; doi:10.1111/jcmm.70925)
Supplement: Supplementary file 2 — Table S2: The association between TYG and different CVD in non‐diabetic hypertension (n = 8109). [file JCMM-29-e70925-s002.docx]

**Supplementary Table 2**：The association between TYG and different CVD in non-diabetic hypertension(n=8109).

| Exposure | HR (95％ CI) P | | | | |
| --- | --- | --- | --- | --- | --- |
|  | CVD death(Events:71) | Heart failure(Events:176) | MI(Events:226) | Stroke(Events:133) | PAD(Events:161) |
| TYG(continuous) | 2.74 (1.64, 4.56)  0.0001 | 1.11 (0.78, 1.57)  0.5756 | 1.37 (1.02, 1.83)  0.0381 | 1.26 (0.85, 1.85)  0.2465 | 1.53 (1.09, 2.16)  0.0137 |
| TYG(quartile) |  |  |  |  |  |
| Q1 | Ref | Ref | Ref | Ref | Ref |
| Q2 | 1.32 (0.63, 2.76)  0.4680 | 1.34 (0.88, 2.04)  0.1714 | 1.15 (0.77, 1.70)  0.5028 | 1.10 (0.66, 1.84)  0.7015 | 1.02 (0.63, 1.67)  0.9228 |
| Q3 | 2.13 (1.03, 4.40)  0.0413 | 1.24 (0.79, 1.95)  0.3542 | 1.47 (0.99, 2.18)  0.0575 | 1.16 (0.69, 1.96)  0.5764 | 1.60 (1.00, 2.57)  0.0502 |
| Q4 | 2.34 (1.04, 5.25)  0.0400 | 1.29 (0.77, 2.15)  0.3280 | 1.35 (0.87, 2.11)  0.1856 | 1.21 (0.69, 2.14)  0.5043 | 1.75 (1.05, 2.94)  0.0333 |
| P for trend | 0.0225 | 0.3874 | 0.1219 | 0.5008 | 0.0137 |

Abbreviations: HR, hazard ratio; CI, confidence interval; Ref, reference; CVD, cardiovascular disease; MI, myocardial infarction；PAD, peripheral arterial disease; CKD, chronic kidney disease; BMI, body mass index

Adjusted for: gender, age, race, smoking status, alcohol consumption, vigorous physical activity, BMI, CKD, aspirin use, statin use, hyperlipidemia, CVD, Framingham risk score.
