# Supplementary material for: Association of Triglyceride–Glucose Index With Different Cardiovascular Diseases in Non‐Diabetic Hypertension
Source: J Cell Mol Med. 2025 Oct 30;29(21):e70925. doi: 10.1111/jcmm.70925 (PMC12573475; doi:10.1111/jcmm.70925)
Supplement: Supplementary file 3 — Table S3: The association between TYG and different CVD in non‐diabetic hypertension. [file JCMM-29-e70925-s001.docx]

**Supplementary Table 3**：The association between TYG and different CVD in non-diabetic hypertension.

| Exposure | HR (95％ CI) P | | | | |
| --- | --- | --- | --- | --- | --- |
|  | CVD death  (Events/N:118/9299) | Heart failure  (Events/N:170/9281) | MI  (Events/N:210/9259) | Stroke  (Events/N:127/9279) | PAD  (Events/N:111/9260) |
| TYG(continuous) | 2.18 (1.48, 3.21)  <0.0001 | 1.22 (0.86, 1.72)  0.2665 | 1.31 (0.98, 1.77)  0.0712 | 1.68 (1.15, 2.44)  0.0067 | 1.47 (0.99, 2.20)  0.0556 |
| TYG(quartile) |  |  |  |  |  |
| Q1 | Ref | Ref | Ref | Ref | Ref |
| Q2 | 1.14 (0.66, 1.96)  0.6461 | 1.11 (0.73, 1.70)  0.6235 | 0.89 (0.58, 1.37)  0.6042 | 1.24 (0.70, 2.20)  0.4673 | 0.81 (0.44, 1.50)  0.5024 |
| Q3 | 1.28 (0.73, 2.25)  0.3933 | 1.03 (0.65, 1.62)  0.9098 | 1.45 (0.97, 2.16)  0.0680 | 1.94 (1.12, 3.34)  0.0175 | 1.46 (0.83, 2.57)  0.1898 |
| Q4 | 2.32 (1.27, 4.23)  0.0060 | 1.37 (0.83, 2.28)  0.2224 | 1.43 (0.90, 2.27)  0.1262 | 2.22 (1.21, 4.07)  0.0096 | 1.73 (0.93, 3.22)  0.0816 |
| P for trend | 0.0055 | 0.2893 | 0.0409 | 0.0040 | 0.0287 |

Abbreviations: HR, hazard ratio; CI, confidence interval; Ref, reference; CVD, cardiovascular disease; MI, myocardial infarction；PAD, peripheral arterial disease; CKD, chronic kidney disease; BMI, body mass index

Adjusted for: gender, age, race, smoking status, alcohol consumption, vigorous physical activity, BMI, CKD, aspirin use, statin use, hyperlipidemia, CVD, Framingham risk score.
